# Supplementary material for: Gut microbiota alterations in postmenopausal women with osteoporosis and osteopenia from Shanghai, China
Source: PeerJ. 2024 May 31;12:e17416. doi: 10.7717/peerj.17416 (PMC11146318; doi:10.7717/peerj.17416)
Supplement: Supplemental Information 3 [file peerj-12-17416-s003.pdf]

Amplicon Sequencing Data Analysis Method

1 Data Analysis

1.1 Raw Data Quality Control

NGS data pre-treatment: According to quality of single nucleotide, raw data was primarily filtered by Trimmomatic[1] (version 0.33). Identification and removal of primer sequences was process by Cutadapt[2] (version 1.9.1). PE reads obtained from previous steps were assembled by USEARCH [4] (version 10) and followed by chimera removal using UCHIME[3] (version 8.1). The high-quality reads generated from above steps were used in the following analysis. Detailed parameter settings in mentioned analysis were shown below.

1) Trimmomatic: Trimmomatic is a trimming tool designed for Illumina sequencing reads. It is applicable to PE reads using phred15 quality score. . For PE reads treatment, forward and reverse fastq files need to be provided. Parameter setting: Window size was set as 36 bp. The reads will be cut from the start of the window once average Q-score within the window is lower than 10.

2) Primer identification and removal: Cutadapt was applied to remove the primer sequences. Parameter setting: Maximum mismatch accepted: 20%; Minimum coverage: 80%. 3) PE reads assembly: PE reads were assembled by Usearch v10. Parameter setting:

3) Minimum length of overlap: 10 bp; Minimum similarity within overlapping region: 90%; Maximum mismatch accepted: 5 bp (Default).

4) Schematic flow of UCHIME was shown in the following figure. Each query sequences is split into non-overlapped chunks. These chunks were compared with reference database to identify the best hit of each chunk in the database and further define two best parent sequences. The query sequence was subsequently compared with the two parent sequences. If a fragment with over 80% similarity to query sequence is found on both parents, this query sequence will be defined as chimera sequence.

1.2 ASV Analysis

ASV Analysis: DADA2[6] method in QIIME2[5](versoin 2020.06) was applied to de-noise sequences, generating ASVs. Conservative threshold for OTU filtration is 0.005%.

1.3 Species Annotation and Taxonomic Analysis

1.3.1 DatabaseSilva [7]（Release138, http://www.arb-silva.de）

1.3.2 Species Annotation

Methods:

1) Blast-based method: Classify-consensus-blast in QIIME2, as the name indicates, is a blast-based annotation method. It identifies the annotation with the highest consensus in N (default: 3) best hits. Parameter setting: Minimum similarity in sequence: 90%; Minimum coverage: 90%; Minimum consensus: 51%.

2) Naive Bayes classifier-based method: Species annotation is processed with classify-sklearn in QIIME. The classifier needs to be trained before use in order to “learn” which features can be used for classification. Parameter setting: Confidence of classifier: 0.7.

3) Combined method: Feature sequences are firstly blast against reference database by classify-consensus-blast. The ones that could not be matched in reference database are further

classified by classify-sklearn.

1.4 Diversity Analysis

1.4.1 Alpha Diversity Analysis

Alpha diversity reflects the species richness of individual sample and the species diversity, there are series of measurement index: Chao1, Ace, Shannon, Simpson. Chao1 and Ace index measure species richness, i.e. the number of species. Shannon and Simpson indexes are used to measure species diversity, they are affected by species richness and community evenness in the sample community. In the case of the same species richness, the higher the evenness of each species in the community is, the higher the community diversity is. Larger Shannon index and smaller Simpson index indicate that the species diversity of the sample is higher. Alpha diversity analysis software: QIIME2（https://qiime2.org/).

1.4.1.1 Rarefaction Curve

Rarefaction Curve was used to verify the sequencing amount which enough to reflect the species diversity in samples. As the sequences count increases within a certain range, the sharp increase of the curve presents that a large number of species were found; when the curve tends to be flat, it means that the species number won’t increase significantly when the sequencing amount increases. Rarefaction curve can be used to determine whether sequencing amount of each sample is sufficient, the sharp increase of the curve means that the sequencing amount is insufficient, more sequencing data is needed; otherwise, the sequencing amount is sufficient for bioinformatics.

1.4.1.2 Shannon Curve

Shannon Curve reflects the index of microbial diversity in samples. The higher the Shannon index is, the more the OTU species are and the richer the species are. It means that the majority of the microbial species information is included in samples. When the curve tends to be flat, it means the sequencing data is sufficient and the OTU species won’t grow with sequencing data growing; if the curve does not tend to be flat, then it means insufficient and more OTUs will be found with more sequencing amount.

1.4.1.3 Rank Abundance Curve Rank

Abundance Curve will rank the OTU abundance of each sample by size and it can represent the relative abundance. The curve explains both the species richness and species evenness in sample. The species richness is reflected by X-axis length of the curve. The wider the curve, the more abundant the composition of species; The species evenness is reflected by the curve shape, the flatter the shape, the more even the composition of species.

1.4.2 Beta Diversity Analysis (n ≥ 3)

Beta diversity analysis to compare the similarity of species diversity between different samples. Beta diversity analysis mainly uses binary jaccard, bray curtis, weighted unifrac (bacteria only), unweighted unifrac (bacteria only) these four algorithms to calculate distance between samples.

1.4.2.1 PCA Analysis

Principal Component Analysis (PCA) is a technique for analyzing and simplifying data sets. By decomposing variances, differences of multiple groups of data are reflected on two-dimensional coordinate graph, the coordinate axis present the two eigenvalues that can reflect the largest variance.

1.4.2.2 PcoA Analysis

Principal Coordinates Analysis(PCoA) is a dimension reduction sorting method similar to PCA, the principle is to assume that there are data that can measure the difference or distance among N samples, then a rectangular coordinate system can be constructed by the method. N samples are replaced by N dots, square of the Euclidean distance between dots equals to the original difference data, so quantitative conversion of data can be realized, the major elements and structure can be extracted from multidimensional data.

1.4.2.3 NMDS Analysis

Non-MetricMulti-Dimensional Scaling (NMDS) is a sorting method suitable for ecological research, it's a data analysis method that can simplify the research objects (samples or variables) from multi-dimensional space to low-dimensional space for positioning, analysis and classification, while retaining the original relationship between objects. Similar to PCA or PCoA, the difference between groups or within groups can be seen from the distribution of samples. The original design of NMDS is to overcome the shortcoming (linear model) of the previous sorting methods including PCA and PCoA. The model of NMDS is non-linear, which can better reflect the non-linear structure of ecological data, so some studies believe that the effect of NMDS is better than PCA/PCoA.

1.4.2.4 UPGMA Analysis

Unweighted Pair-group Method with Arithmetic Mean (UPGMA) is a commonly used clustering analysis method for sample hierarchical clustering. The principle is to assume that number of divergences occur in each lineage is the same, which means the replacement rate of nucleotides or amino acids is equal and constant. The phylogenetic tree generated by UPGMA method can be said to be a simple embodiment of the species tree. After each divergence, the length of branches from the common ancestor node to the two OTUs is the
same.

1.4.2.5 Sample Heatmap Analysis

Heatmap can get the distance matrix between samples by using distance algorithms(binary, bray, weighted, unweighted), heatmap of samples is drawn by using R language tool, the difference between two samples can be visually seen according to the change of color gradient.

1.4.2.6 Anosim and Adonis Analysis

PERMANOVA (Adonis) is known as Displacement multivariate analysis of variance, Anosim(analysis of similarities) is known as similarity analysis, they are statistical methods mainly used to analyze the similarity between multi-dimensional data groups. In PCoA analysis and NMDS analysis, there is no corresponding statistical test conclusion on whether the difference between different groups of samples is significant. PERMANOVA or Anosim analysis can test whether there is a significant difference in beta diversity between samples from different groups. Use vegan pack in R language to do analysis and python to plot. R2 obtained by PERMANOVA analysis represents the interpretation degree of sample difference between different groups -- the ratio of group variance to total variance. A larger R2 indicates that grouping has a higher interpretation degree to the difference, the group difference is higher, and P value less than 0.05 indicates a high reliability of the test. The closer the R value(obtained from Anosim analysis) is to 1, the higher the difference between groups is than that within groups; the smaller the R value is, the less significant difference is between them; P value less than 0.05 indicates high reliability of the test.

1.5 Significant Difference Analysis

1.5.1 LefSe Analysis

LefSe (LDA Effect Size) is An algorithm for High-Dimensional biomarker discovery and explanation that identifies genomic features (genes, pathways, or taxa) characterizing the differences between two or more biological conditions. It emphasizes both statistical significance and biological relevance, allowing researchers to identify differentially abundant features that are also consistent with biologically meaningful categories (subclasses). LEfSe first robustly identifies features that are statistically different among biological classes. It then performs additional tests to assess whether these differences are consistent with respect to expected biological behavior. we first use the non-parametric factorial Kruskal-Wallis (KW) sum-rank test to detect features with significant differential abundance with respect to the class of interest; biological significance is subsequently investigated using a set of pairwise tests among subclasses using the (unpaired) Wilcoxon rank-sum test. As a last step, LEfSe uses Linear Discriminant Analysis to estimate the effect size of each differentially abundant feature and, if desired by the investigator, to perform dimension reduction.

1.6 Functional Prediction

1.6.1 PICRUSt2 Function Prediction

PICRUSt software was used to predict functional genes composition in the samples by comparing species composition information obtained from 16S sequencing data, then to analyze functional differences between samples or groups.

Note: If a certain analysis is not signed in contract or the sample is not suitable for a certain analysis, the final reports and result files will not provide corresponding results.

【References】

1. Bolger AM, Lohse M, Usadel B: Trimmomatic: a flexible trimmer for Illumina sequence data. Bioinformatics 2014:btu170. Bolger AM, Lohse M, Usadel B: Trimmomatic: a flexible trimmer for Illumina sequence data. Bioinformatics 2014:btu170.

2. Martin M.MartinM.Cut adapt removes adapter sequences from high-throughput sequencing reads, EMBnet. EMBnet 17:10-12[J]. Embnet Journal, 2011, 17(1).

3. Edgar RC, Haas BJ, Clemente JC, Quince C, Knight R: UCHIME improves sensitivity and speed of chimera detection. Bioinformatics 2011, 27(16):2194-2200.

4. Edgar Robert C. UPARSE: highly accurate OTU sequences from microbial amplicon reads.[J] .Nat. Methods, 2013, 10(10): 996-8.

5. Bolyen E, Rideout JR, Dillon MR, Bokulich NA, Abnet CC, Al-Ghalith GA, Alexander H, Alm EJ, Arumugam M, Asnicar F, Bai Y, Bisanz JE, Bittinger K, Brejnrod A, Brislawn CJ, Brown CT, Callahan BJ, Caraballo-Rodríguez AM, Chase J, Cope EK, Da Silva R, Diener C, Dorrestein PC, Douglas GM, Durall DM, Duvallet C, Edwardson CF, Ernst M, Estaki M, Fouquier J, Gauglitz JM, Gibbons SM, Gibson DL, Gonzalez A, Gorlick K, Guo J, Hillmann B, Holmes S, Holste H, Huttenhower C, Huttley GA, Janssen S, Jarmusch AK, Jiang L, Kaehler BD, Kang KB, Keefe CR, Keim P, Kelley ST, Knights D, Koester I, Kosciolek T, Kreps J, Langille MGI, Lee J, Ley R, Liu YX, Loftfield E, Lozupone C, Maher M, Marotz C, Martin BD, McDonald D, McIver LJ, Melnik AV, Metcalf JL, Morgan SC, Morton JT,

Naimey AT, Navas-Molina JA, Nothias LF, Orchanian SB, Pearson T, Peoples SL, Petras D, Preuss ML, Pruesse E, Rasmussen LB, Rivers A, Robeson MS, Rosenthal P, Segata N, Shaffer M, Shiffer A, Sinha R, Song SJ, Spear JR, Swafford AD, Thompson LR, Torres PJ, Trinh P, TripathiA, Turnbaugh PJ, Ul-Hasan S, van der Hooft JJJ, Vargas F, Vázquez-Baeza Y, Vogtmann E, von Hippel M, Walters W, Wan Y, Wang M, Warren J, Weber KC, Williamson CHD, Willis AD, Xu ZZ, Zaneveld JR, Zhang Y, Zhu Q, Knight R, and Caporaso JG. 2019. Reproducible, interactive, scalable and extensible microbiome data science using QIIME 2. Nature Biotechnology 37: 852–857.

6. Callahan, B., McMurdie, P., Rosen, M. et al. DADA2: High-resolution sample inference from Illumina amplicon data. Nat Methods 13, 581–583 (2016). https://doi.org/10.1038/nmeth.3869.

7. Quast C, Pruesse E, Yilmaz P, Gerken J, Schweer T, Yarza P, Peplies J, Glöckner FO (2013) The SILVA ribosomal RNA gene database project: improved data processing and web-based tools.
